# *Nyctanthes arbor-tristis* Ameliorated FCA-Induced Experimental Arthritis: A Comparative Study among Different Extracts

**DOI:** 10.1155/2017/4634853

**Published:** 2017-06-06

**Authors:** Maliha Uroos, Zaigham Abbas, Shumaila Sattar, Nigarish Umer, Arham Shabbir, Shafiq-ur-Rehman, Ahsan Sharif

**Affiliations:** ^1^Institute of Chemistry, University of the Punjab, Lahore, Punjab 54590, Pakistan; ^2^Department of Microbiology and Molecular Genetics, University of the Punjab, Lahore, Punjab 54590, Pakistan; ^3^Pharmacology Section, Faculty of Pharmacy, The University of Lahore, Lahore, Punjab, Pakistan

## Abstract

*Nyctanthes arbor-tristis* (NAT) is commonly used traditionally for the treatment of rheumatism and inflammatory diseases. Current study evaluates the antiarthritic potential of NAT using Freund's adjuvant-induced arthritic rat model. Treatments with methanolic, ethyl acetate, and n-hexane extracts were continued for consecutive 20 days. Macroscopic arthritic scoring and water displacement plethysmometry were used to evaluate arthritic development. Hematological and biochemical parameters were investigated and ankle joints were processed for histopathological evaluation. Qualitative phytochemical analysis and GC-MS analysis were conducted for identification of constituents. NAT extracts suppressed arthritic scoring, paw edema, infiltration of inflammatory cells, pannus formation, and bone erosion. The plant extracts ameliorated total leukocytes and platelet counts and nearly normalized red blood cells (RBC) counts and hemoglobin (Hb) content. The extracts were found safe in terms of hepatotoxicity and nephrotoxicity as determined by aspartate aminotransferase (AST), alanine aminotransferase (ALT), creatinine, and urea levels. Comparative analysis showed that ethyl acetate extract produced the highest inhibition of paw edema. The major constituents found in ethyl acetate extract can be classified into three major classes, that is, terpenes, terpenoids, fatty acids, and iridoid glycosides. Current study showed that* Nyctanthes arbor-tristis* ameliorated experimental rheumatoid arthritis and ethyl acetate extract possessed the highest inhibitory activity.

## 1. Introduction

Rheumatoid arthritis (RA) is characterized by chronic inflammation which results in impaired movement and disability. It is an autoimmune condition of synovial joints which is triggered generally by inflammatory mediators and infections [1]. The standard mortality ratio with RA differs from 1.28 to 2.89 and the data from the adult population of developed countries show approximate prevalence rate of 0.5% to 1% [2].

Current antiarthritic regimen includes nonbiologic disease modifying antirheumatic drugs (DMARD), such as methotrexate; nonsteroidal anti-inflammatory drugs (NSAID), such as piroxicam; biological therapies, such as inhibitors of IL-6, IL-1, and TNF-*α*; and glucocorticoids, such as methyl prednisolone and triamcinolone [3–6]. Although NSAIDS are still considered as “first-line” therapy of RA and they effectively attenuate swelling, pain, and joint stiffness, yet there role is limited in changing the course of disease [7–9]. Most of the NSAIDs are associated with adverse reactions including stomach ulcers, bleeding, dyspepsia, and risk of cardiovascular problems, while treatment with nonbiologic DMARDs can cause reversible alopecia, rash, nausea, loss of appetite, elevated formation of rheumatoid nodules, mouth ulcers, and neurological problems. Anti-TNFs therapy is also related to abdominal pain, headache, itching, bruising, bleeding, rash, vomiting, diarrhea, injection site reaction, cellulitis, and respiratory tract infections. Chronic use of glucocorticoids leads to multiple adverse effects which include increase risk of osteoporosis, diabetes mellitus, peptic ulcer, gastrointestinal bleeding, cataracts, and infections [3]. Due to persisting symptoms and associated adverse effects with the existing therapy, increasing percentage of patients with RA is returning to complementary and alternative medicine [10, 11].

*Nyctanthes arbor-tristis *(NAT) Linn (Family: Oleaceae) is known to emit pleasant and strong fragrance during the whole night, hence popularly called “Harsinghar” in Hindi and “Night Jasmine” in English [12, 13]. The leave juice and decoction are commonly used in traditional system of medicine to treat rheumatism, arthritis, and inflammatory disorders. Previously, pharmacological evaluation showed that NAT possessed anti-inflammatory, antimalarial, antioxidant, antimicrobial, antifungal, antidiabetic, anticancer, antiviral, immunostimulant, hepatoprotective, and CNS depressant activities (reviewed in [14, 15]).

Current study was aimed at validating the traditional uses of NAT in rheumatism and arthritis. Antiarthritic activity was evaluated using rat model of FCA-induced arthritis. Other objectives of the study were to compare the antiarthritic activities of different plant extracts with each other and also with piroxicam, a commonly used reference drug. Present study also tended to identify the constituents of ethyl acetate extract using qualitative phytochemical analysis and GC-MS analysis.

## 2. Materials and Methods

### 2.1. Plant Material and Preparation of Methanol, Ethyl Acetate, and n-Hexane Extracts

Mature leaves of the plant (NAT) were collected in the month of November, 2015, from University of the Punjab, Lahore. The plant was identified by Dr. Abdul Rehman Khan Niazi, in-charge herbarium, University of the Punjab, Lahore, and a specimen voucher (LAH # 90217) was deposited in the herbarium. The plant leaves were washed and dried for 15 days under shade. Leaves were grinded and fine powder was collected. 500 grams of leave powder was immersed in 2 L methanol and placed for 10 days with daily occasional shaking. All the material was initially filtered through muslin cloth and subsequently through filter paper (Whatman number 1). Greenish black colored filtrate was obtained which was evaporated on a rotary evaporator under reduced pressure to a semisolid thick extract [16]. The approximate percentage yield was calculated as 19%.

50 g of crude methanol extract was dissolved in 500 ml distilled water. The solution was subjected to liquid-liquid extraction with organic solvent n-hexane using separating funnel. N-Hexane fraction was collected and solvent was evaporated using rotary evaporator under reduced pressure. The resultantly obtained yield was calculated as 13.5%. After separation of n-hexane layer, aqueous layer was again subjected to fractionation with ethyl acetate. Similar procedure was used as described in preparation of n-hexane fraction. The resultantly obtained yield was determined as 30%.

### 2.2. Housing of Animals

Sprague Dawley rats, aging 6–8 weeks, of either sex, weighing 150–250 grams were used for the study. Before the beginning of experiment, all the rats were kept in the animal house of Faculty of Pharmacy, University of Lahore, for a period of 1 week in order to acclimatize them to the environment. Animals were given tap water and standard diet and were kept at 12 hours dark/light cycles. Standard conditions of humidity (40–60%) and temperature (24–26°C) were maintained [17]. The study was approved by the Institutional Animal Ethics Committee, University of Lahore (IAEC-2015-11A).

### 2.3. Experimental Design

A total of 36 rats were used which were divided into 6 groups. Each group contained 6 rats (3 males and 3 females). The grouping and dosing schedule was as follows [18–20].

*Negative Control*. Rats were treated with normal saline only.

*Arthritic Control*. Arthritis was induced in rats with FCA and treated with normal saline only.

*Methanol Extract Treated (NA Meth)*. Arthritic rats were treated with methanol extract (500 mg/kg b.w.) orally for 20 consecutive days (from day 8 to day 27).

*n-Hexane Extract Treated (NA Hex)*. Arthritic rats were treated with n-hexane extract (500 mg/kg b.w.) orally for 20 consecutive days (from day 8 to day 27).

*Ethyl Acetate Extract Treated (NA Ethacet)*. Ethyl acetate extract (500 mg/kg b.w.) was given to the arthritic rats orally for 20 consecutive days (from day 8 to day 27).

*Reference Drug Treated (Piroxicam)*. The rats were intraperitoneally injected with piroxicam (10 mg/kg b.w.). The duration of treatment was similar to other extract treated groups.

### 2.4. Induction of Arthritis

Arthritis was induced by administration of Complete Freund's Adjuvant (Sigma-Aldrich, Lot# F5881) that was comprised of 1 mg of heat-killed and dried* Mycobacterium tuberculosis* (strain H37Ra, ATCC 25177), dissolved in 0.85 ml paraffin oil and 0.15 ml of mannide monooleate. 0.15 ml quantity of CFA was injected at subplantar region of the right posterior paws of the rats. On day 0, CFA was injected in all the groups except negative control group. The treatment was commenced at 8th day of arthritic induction and all animals were sacrificed at day 28 [21].

### 2.5. Evaluation of Arthritic Development

The occurrence and rigorousness of arthritis were determined by macroscopic arthritic scoring method. The rats were examined for the presence of detectable signs that were categorized by edema and erythema in the posterior paws of the rats. The scoring was conducted at days 12, 16, 20, 24, and 28 using the following macroscopic standards: 0 was given to normal paw and similarly scores 1 to 4 were related to edema and erythema of a single digit to the involvement of complete paw or entire digit [21].

### 2.6. Evaluation of Paw Edema Using Plethysmometer

Plethysmometer was used to evaluate the paw volume (as an indicator of edema) of the rats at day 28 using water displacement technique. Paw volumes of both right and left hind paws were evaluated where left paw served as a negative control. The increase in paw edema was evaluated by determining the difference between the paw volumes of ipsilateral (injected paw) and contralateral (noninjected paw) paws [5]. The results were also expressed as percentage inhibition using following formula:

(1) $$\begin{array}{c} \%\text{ inhibition}=\frac{\text{Mean paw volume of positive control group}-\text{Mean paw volume of treated group}}{\text{Mean paw volume of positive control group}} \end{array}$$

### 2.7. Histopathological Evaluation of Ankle Joints

The ankle joints were horizontally cut and tissues were fixed in 10% buffered formalin. All the tissues were subsequently decalcified using 10% formic acid dissolved in formalin. Then, the samples were subjected to further processing steps including dehydration and paraffin embedding. Blocks were solidified and 0.5 *μ*m thick tissue sections were cut. All the slides were stained with H & E stains and results were semiquantified. A blind histopathologist examined the bone erosion, pannus formation, and infiltration of inflammatory cells by using following scale: 0, normal; 1, mild; 2, moderate; and 3, severe [20].

### 2.8. Hematological Analysis

Different types of hematological parameters like total leukocyte counts (TLC), red blood cell (RBC) counts, platelets counts, and Hb content were determined using automated hemocytometer. The blood samples were collected from the rats using cardiac puncture technique.

### 2.9. Liver and Renal Function Tests

ALT and AST levels were determined to evaluate the possible hepatotoxic effects of the plant extracts, while urea and creatinine levels were measured to assess the possible nephrotoxic effects. Kit manufacturer's protocol was followed and experiments were conducted using automated chemistry analyzer.

### 2.10. Qualitative Phytochemical Analysis of Ethyl Acetate Extract

Phytochemical screening of the ethyl acetate extract was performed qualitatively for the possible presence of alkaloids, carbohydrates, flavonoids, glycosides, tannins, terpenoids, acids, coumarins, carotenoids, and phenols [22–24].

### 2.11. GC-MS Analysis

Extracts solutions were made by dissolving 2 mg of solid extract in 10 ml ethyl acetate for GC-MS analysis. GC-MS conditions for the analysis of NAT leaves extracts used were as follows: capillary column # DB-5MS (30 m × 250 *µ*m × 0.25 *µ*m film thickness), helium as a carrier gas with 1 mL/min flow rate, and 1 *µ*L injection volume of extract solution in splitless mode. Oven operating conditions: initially 60°C, then increasing at a rate of 10°C/min, and finally 300°C for 5 min.

Mass spectrometer conditions: 250°C ion source temperature, 04 min solvent delay, 70 ev ionizing voltage, 20 to 800 m/z range, and 200°C mass quadrupole analyzer temperature. Total run time of extract solution was 28 min.

### 2.12. Statistical Analysis

Mean ± SEM was used for data representation; the latter comprised 06 readings per group. One-Way ANOVA followed by Tukey's multiple comparison test/Student's *t*-test was applied to analyze the data, where applicable. *P* < 0.05 was considered as statistically significant.

## 3. Results

### 3.1. NAT Inhibited Development of Rheumatoid Arthritis

**Figure 1:**
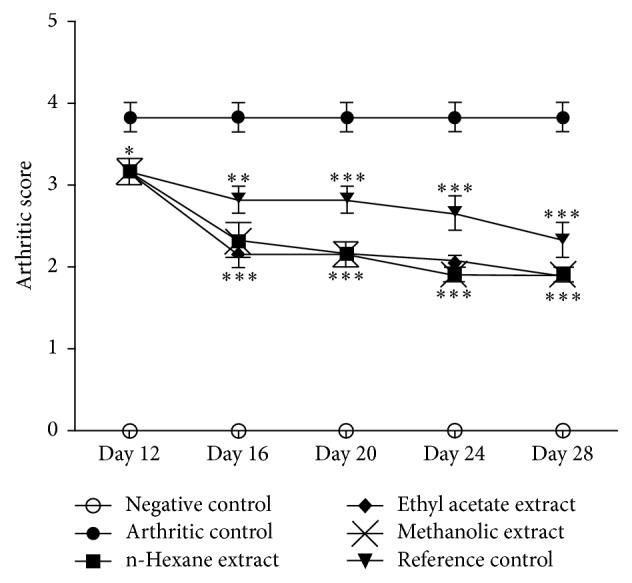
*N. arbor-tristis* significantly inhibited arthritic development as determined by macroscopic arthritic scoring at days 12, 16, 20, 24, and 28. Mean ± SEM is used for data representation, where *n* = 6. ^*∗*^*P* < 0.05, ^*∗∗*^*P* < 0.01, and ^*∗∗∗*^*P* < 0.001.

Signs and lesions of rheumatoid arthritis were observed in injected paw which represented primary lesions. Inflammatory edema was observed in unilateral paw around days 1–8 caused by the administration of FCA. Rats started receiving treatment at 8th day of arthritic induction and significant (*P* < 0.05) attenuation of arthritic development was observed at 12th day in all groups. At the last day of arthritic treatment, that is, day 28, all the treated groups showed similar suppression of macroscopic arthritic score (1.917 ± 0.0833 each; *P* < 0.001) as compared with arthritic control group (3.833 ± 0.166). Treatment with piroxicam also significantly inhibited arthritic development (2.333 ± 0.210; *P* < 0.001) as compared with arthritic control group (Figure 1).

### 3.2. Treatment with NAT Significantly Suppressed Paw Edema

**Figure 2:**
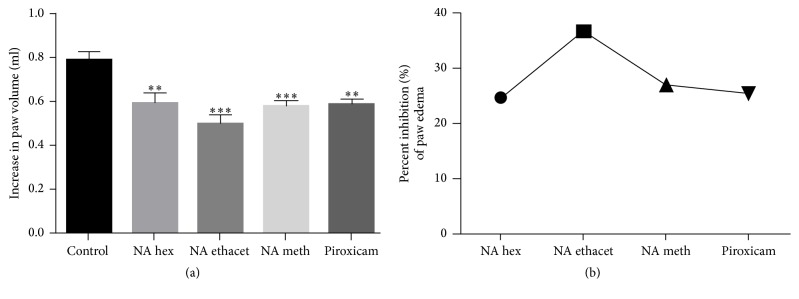
Plethysmometeric analysis showed that* N. arbor-tristis* significantly suppressed paw edema (a) and ethyl acetate extract exhibited the highest percentage suppression (b). Mean ± SEM is used for data representation, where *n* = 6. ^*∗∗*^*P* < 0.01, ^*∗∗∗*^*P* < 0.001.

Paw edema was measured using water displacement plethysmometer at day 28. We found significant suppression of paw edema after treatment with n-hexane (0.5967 ± 0.0435; *P* < 0.01), ethyl acetate (0.5017 ± 0.0380; *P* < 0.001), and methanol (0.5783 ± 0.0245; *P* < 0.001) extracts as compared with arthritic control group (0.7917 ± 0.0322). Treatment with piroxicam (0.5900 ± 0.0208; *P* < 0.01) also showed significant reduction in paw edema as compared with arthritic control group (Figure 2(a)).

The results were also expressed in terms of percentage inhibition, which showed that n-hexane (24.63%), ethyl acetate (36.63%), and methanol (26.95%) extracts markedly inhibited paw edema. Piroxicam also provided 25.47% inhibition of paw edema when compared with control group (Figure 2(b)).

### 3.3. Treatment with NAT Significantly Attenuated Inflammatory Cell Infiltration

**Figure 3:**
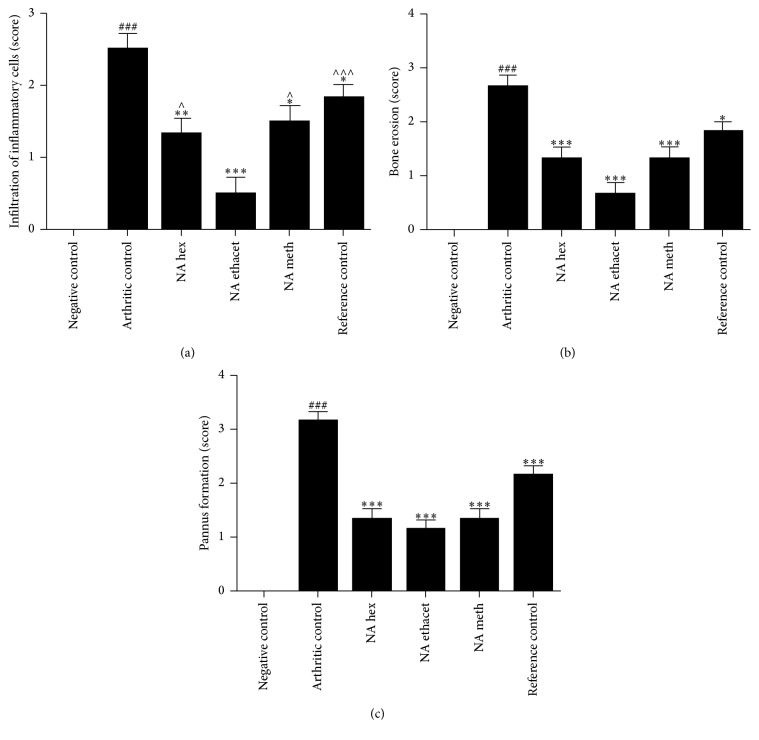
Treatment with* N. arbor-tristis* extracts ameliorated infiltration of inflammatory cells (a), bone erosion (b), and pannus formation (c). Mean ± SEM is used for data representation, where *n* = 6. ^*∗*^*P* < 0.05, ^*∗∗*^*P* < 0.01, and ^*∗∗∗*^*P* < 0.001. Symbol “∧” represents the comparison of ethyl acetate extract with other treated groups. # and ### indicate the comparison of negative control with arthritic control.

**Figure 4:**
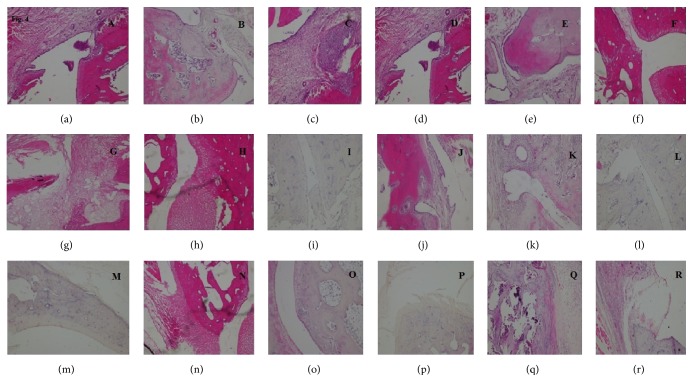
Representative microscopic figures of histopathological analysis (H&E). Figures (a–f), (g–l), and (m–r) represent negative control, arthritic control, n-hexane extract, ethyl acetate extract, methanolic extract, and reference control, respectively. Infiltration of inflammatory cells is represented by Figures (a–f), pannus formation is represented by Figures (g–l), and bone erosion is represented by Figures (m–r).

Inflammatory cell infiltration was determined using histopathological scoring method. We found significant elevation in inflammatory cell infiltration in arthritic control (2.5 ± 0.223; *P* < 0.001) group as compared with negative control group. All three extracts, that is, n-hexane (1.333 ± 0.210; *P* < 0.01), ethyl acetate (0.5 ± 0.223; *P* < 0.001), and methanol (1.5 ± 0.223; *P* < 0.05) extracts, significantly attenuated inflammatory cell infiltration as compared with arthritic control group. When compared with each other, ethyl acetate extract showed significantly higher suppression of inflammation as compared with other treated groups. Piroxicam also significantly (1.833 ± 0.1667) decreased inflammation as compared with arthritic control group (Figures 3(a) and 4(a)–4(f)).

### 3.4. Treatment with NAT Caused Significant Reduction in Bone Erosion

The data showed significant increase in bone erosion in arthritic control group (2.667 ± 0.2108; *P* < 0.001) as compared with negative control group. Treatment with n-hexane (1.333 ± 0.2108), ethyl acetate (0.6667 ± 0.2108), and methanol (1.333 ± 0.2108) extracts significantly (*P* < 0.001) inhibited bone erosion as compared with arthritic control group. Similarly, piroxicam also significantly (1.833 ± 0.1667; *P* < 0.05) reduced bone erosion as compared with arthritic control group. When treated groups were compared with each other, ethyl acetate extract showed significantly (*P* < 0.01) higher inhibition as compared with reference control group (Figures 3(b) and 4(m)–4(r)).

### 3.5. NAT Significantly Decreased Pannus Formation

Pannus formation was found significantly increased in arthritic control group (3.167 ± 0.1667; *P* < 0.001) as compared with negative control group. The data showed significant reduction in pannus formation after treatment with all three plant extracts. Treatment with n-hexane (1.333 ± 0.2108), ethyl acetate (1.167 ± 0.1667), and methanol (1.333 ± 0.2108) extracts significantly (*P* < 0.001) attenuated pannus formation as compared with arthritic control group. Treatment with piroxicam also significantly (2.167 ± 0.1667) suppressed pannus formation as compared with arthritic control group (Figures 3(c) and 4(g)–4(l)).

### 3.6. Treatment with NAT Significantly Attenuated the Elevated Total Leukocyte and Platelet Counts

TLC levels were found significantly raised in arthritic control group (8.170 ± 0.7533; *P* < 0.001) as compared with negative control group (3.108 ± 0.344). Treatment with n-hexane (5.338 ± 0.485; *P* < 0.01), ethyl acetate (3.992 ± 0.254; *P* < 0.001), and methanol (5.452 ± 0.596; *P* < 0.01) extracts significantly reduced the TLC as compared with arthritic control group. The data showed that ethyl acetate extract nearly normalized the TLC and produced significantly (*P* < 0.05) higher improvement as compared with other extract treated groups.

**Figure 5:**
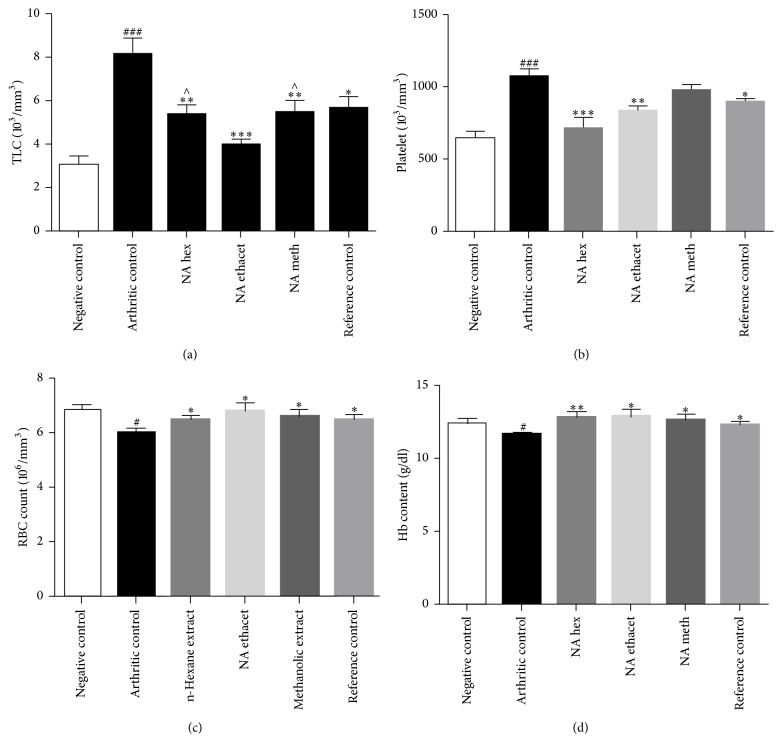
The data showed significant reduction in elevated counts of total leukocytes and platelets (a, b). Treatment with* N. arbor-tristis* extracts also nearly normalized RBC counts and Hb content (c, d). Mean ± SEM is used for data representation, where *n* = 6. ^*∗*^*P* < 0.05, ^*∗∗*^*P* < 0.01, and ^*∗∗∗*^*P* < 0.001. Symbol “∧” represents the comparison of ethyl acetate extract with other treated groups. # and ### indicate the comparison of negative control with arthritic control.

Similarly, we found significantly (*P* < 0.001) elevated platelet levels in arthritic control group (1072 ± 54.28) as compared with negative control group (650.3 ± 40.35). Methanol extract (983 ± 31.40) showed insignificant reduction when compared with arthritic control group. Treatment with n-hexane extract (715.7 ± 73.06; *P* < 0.001), methanol extract (838.2 ± 25.18; *P* < 0.01), and piroxicam (901.3 ± 20.22; *P* < 0.05) caused significant attenuation of platelet counts as compared with arthritic control group (Figures 5(a) and 5(b)).

### 3.7. NAT Nearly Normalized the RBC Counts and Hb Content

The data revealed significantly (*P* < 0.05) reduced RBC counts in arthritic control group (6.012 ± 0.1638) as compared with negative control group (6.822 ± 0.2062). Treatment with n-hexane (6.53 ± 0.1162), ethyl acetate (6.83 ± 0.2826), and methanol extracts (6.632 ± 0.1931) nearly normalized the RBC counts as compared (*P* < 0.05) with arthritic control group. Piroxicam treated group (6.535 ± 0.1196) also showed significant (*P* < 0.05) elevation as compared with arthritic control group.

Similarly, Hb content was found significantly (*P* < 0.05) reduced in arthritic control group (11.70 ± 0.174) as compared with negative control group (12.48 ± 0.2822). The results showed normalization of Hb content after treatment with n-hexane (12.89 ± 0.3101; *P* < 0.01), ethyl acetate (12.92 ± 0.4764; *P* < 0.05), and methanol (12.68 ± 0.3694) extracts. Treatment with piroxicam (12.33 ± 0.2124) also nearly normalized Hb content (Figures 5(c) and 5(d)).

### 3.8. NAT Showed No Clinical Signs of Nephrotoxicity and Hepatotoxicity

**Figure 6:**
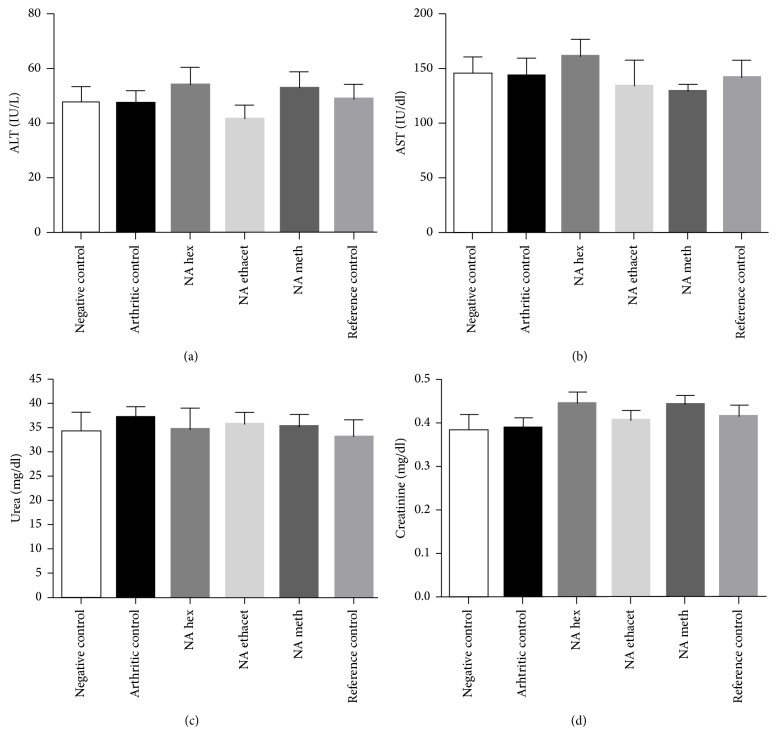
*N. arbor-tristis *extracts were found safe in terms of hepatotoxicity and nephrotoxicity as nonsignificant difference was found among the levels of ALT (a), AST (b), urea (c), and creatinine (d). Mean ± SEM is used for data representation, where *n* = 6.

ALT and AST levels were determined to evaluate the possible hepatotoxic effects of the plant extracts. The data showed no significant difference among all groups. Similarly, urea and creatinine levels were evaluated to find out possible nephrotoxic effects of* N. arbor-tristis* extracts. The extracts were found safe in terms of the clinical signs of nephrotoxicity as the difference among all groups was found statistically nonsignificant (Figures 6(a)–6(d)).

The ethyl acetate extract of NAT depicted the highest antiarthritic activity and, therefore, the phytochemical profile of the extract was analyzed using qualitative test and GC-MS analysis.

### 3.9. Qualitative Phytochemical Analysis

Phytochemicals tests revealed the presence of various constituents which are mentioned in Table 1.

### 3.10. GC-MS Analysis

**Figure 7:**
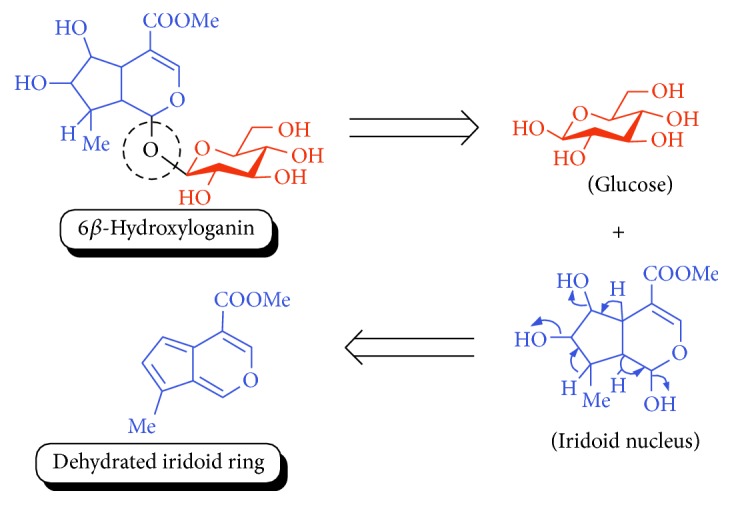
Plausible mechanism for the formation of iridoid ring from iridoid glycoside under GC-MS conditions.

List of all identified components of ethyl acetate extract from mass chromatogram along with their chemical structures, % peak area, and retention time is mentioned in Table 2. GC-MS revealed the presence of glucose and cyclopenta[c]pyran-7-methyl-4-carboxy-methyl ester, which is a dehydrated form of iridoid nucleus of iridoid glycosides, often referred to as 6*β*-hydroxyloganin. It is plausible that, under GC-MS conditions (high temperature~300°C), the iridoid glycoside may have split into its components and subsequently dehydrated with loss of three water molecules. The proposed mechanism for the formation of dehydrated iridoid ring from iridoid glycoside is given in Figure 7.

## 4. Discussion

Current study investigated the antiarthritic potential of different extracts of* N. arbor-tristis* leaves. The data showed that all the extracts possessed antiarthritic activities, while ethyl acetate extract demonstrated the highest inhibition of paw edema. GC-MS analysis revealed the presence of terpenes, terpenoids, fatty acids, and iridoid glycosides in ethyl acetate extract. RA is one of the commonest systemic autoimmune diseases characterized by chronic and progressive inflammation of synovial joints that leads to the pain; inflammation of synovial membrane; pannus formation; cartilage rupture; deformity, destruction, and disability of joints; and premature death in most patients. About 1% of the total world's population is affected by RA and its prevalence is three times more common in women as compared with men [25, 26]. The pathological and clinical changes associated with FCA-induced arthritis are comparable with those found in human RA, making it the most commonly used chronic test model [27].

The data showed that NAT significantly attenuated inflammatory paw edema which is in line with the inferences of histopathological evaluation of ankle joints showing reduced infiltration of inflammatory cells. Chronic inflammation of synovial tissue leads to the deformity of cartilage and bone [28]. We also found significant attenuation of raised bone erosion after treatment with the plant extracts, which might be attributed to the suppression of infiltration of inflammatory cells by NAT extracts.

RA is also associated with multiple hematological alterations. We found high total leukocyte counts and platelet counts in blood which were significantly reduced after treatment as compared with arthritic control group. The induction of immune system against pathogen attack is considered as the major causative factor for elevation in blood leukocyte counts [29]. One of the pathologies of clinical importance in RA is incorrect resolution of inflammation. Leukocyte infiltration in the synovial compartment results in the development of synovial inflammation. The cells which are thought to infiltrate include macrophages, granulocytes, CD8^+^ and CD4^+^ type T cells, B cells, and natural killer cells. All these cells generate huge quantity of proinflammatory cytokines and chemokines [30]. The data exhibited reduced RBC counts and Hb content in arthritic control group which is suggestive of anemic state. Treatment with NAT extracts nearly normalized the altered hematological parameters. Anemia is one of the major hematological manifestations and can be caused by multiple factors, such as ineffective erythropoiesis, bone marrow depression, disease activity, nutritional problems, gastrointestinal bleeding, and drug induction [31].

We also evaluated the possible hepatotoxic and nephrotoxic effects of plant extracts. The data showed the absence of clinical signs of hepatotoxic and nephrotoxic effects in groups treated by the plant extracts. The safety of* N. arbor-tristis* is reflected by the nonsignificant difference of urea, creatinine, ALT, and AST levels among all groups.

Comparative analysis revealed that ethyl acetate extract showed more promising antiarthritic activity as compared with other extracts. It produced the highest inhibition (36.63%) of paw edema as compared with n-hexane and methanolic extracts. Ethyl acetate extract caused more significant (*P* < 0.05) attenuation of infiltration of inflammatory cells in ankle joint and total leukocyte count in blood as compared with other extracts and reference drug. Treatment with ethyl acetate extract also showed more significant (*P* < 0.05) reduction of bone erosion as compared with piroxicam. Therefore, we selected ethyl acetate extract for further qualitative phytochemical screening and identification of components. The major constituents found in ethyl acetate extract can be classified into three major classes, that is, terpenes and terpenoids, fatty acids, and iridoid glycosides. The results of current study showed the presence of phytol, eugenol, and *α*-terpineol in ethyl acetate extract. Phytol plays anti-inflammatory role by suppressing oxidative stress and cytokine production [32]. Eugenol has been demonstrated to possess anti-inflammatory activity via regulating redox reaction [33]. *α*-Terpineol is another known anti-inflammatory phytochemical constituent, which inhibits proinflammatory IL-6 receptor gene expression levels [34]. Terpenoids are also known to regulate inflammatory and immune responses by inhibiting nuclear factor kappaB [35]. Therefore, the antiarthritic activity demonstrated by NAT might be attributed to the presence of these terpenes and terpenoids. Previously, a few other terpenes have also been reported to possess anti-inflammatory properties, such as sugiol, limonene, *α*-pinene, citral, citronellal, and *β*-caryophyllene [36].

The other phytochemicals identified by GC-MS analysis were benzoic acid, cinnamic acid, glucose, and dehydrated iridoid ring. Iridoids are cyclopanta[c]pyran monoterpenoids, often bound to glucose and referred to as iridoid glycosides or loganin.* N. arbor-tristis* leaves extracts are reported to have benzoic and cinnamic acid esters of iridoid glycosides. Previously, the following iridoid glycosides have been identified from the leave extract of* N. arbor-tristis*: arborside-A, arborside-B, arborside-C isoarborside-C, 6- and 7-O-trans-cinnamoyl-6*β*-hydroxyloganin [37–39]. Iridoid ring has been reported to possess anti-inflammatory activity like NSAIDs. The methyl ester moiety on iridoid ring of loganin increases its anti-inflammatory activity. Iridoid ring of loganin is potent inhibitor of proinflammatory cytokines secretion like TNF-*α*, NF-*κ*B, IL-1, and IL-16. It also inhibits the activity of cyclooxygenase enzyme (COX-2) and suppressed the prostaglandins (PGE2) synthesis [40, 41].

## 5. Conclusions

Current study validated the traditional use of* N. arbor-tristis* in arthritis, rheumatism, and inflammatory disorders. The data showed that NAT extracts possessed antiarthritic property which was evident by inhibition of arthritic development during the course of treatment. These inferences were further validated by suppression of paw edema, infiltration of inflammatory cells, bone erosion, and pannus formation found in this study. Treatment with NAT nearly normalized hematological parameters and was found safe in terms of hepatotoxicity and nephrotoxicity. Ethyl acetate extract showed the highest inhibition of paw edema among all extracts. Terpenes, terpenoids, fatty acids, and iridoid glycosides were majorly identified constituents in ethyl acetate extract. The antiarthritic activity might be attributed to the presence of these phytochemical constituents; however, further studies are required for isolation and confirmation of pharmacological activity.

## Figures and Tables

**Table 1:** Qualitative phytochemical analysis of NAT ethyl acetate extract.

Sr. number	Phytochemicals	Test name	Ethyl acetate extract
1	Alkaloids	Hager's test	−
2	Carbohydrates	Benedict's test	+
3	Flavonoids	NaOH test Jone's test	−
4	Glycoside	Foam test	+
5	Tannins	Ferric chloride and KMnO_4_ test	+
6	Terpenoids	Salkowski test	+
7	Acids	Bicarbonates	+
8	Coumarins	Tests for coumarins	−
9	Carotenoids	Test for carotenoids	−
10	Phenols	FeCl_3_ test	+

**Table 2:** List of identified components of ethyl acetate extract from mass chromatograms.

Sr. number	Retention time (min)	% of total	Name of identified components	Molecular formula	Molecular weight (g/mol)	Structures
1	7.170	6.317	Benzoic acid	C_7_H_6_O_2_	122	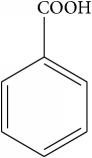

2	7.710	5.496	*α*-Terpineol	C_10_H_18_O	154	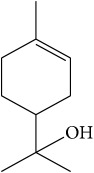

3	11.707	2.373	Glucose	C_6_H_12_O_6_	180	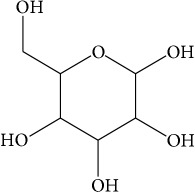

4	12.918	1.931	Cyclopenta[c]pyran-7-methyl-4-carboxy-methyl ester (dehydrated iridoid ring)	C_11_H_10_O_3_	190	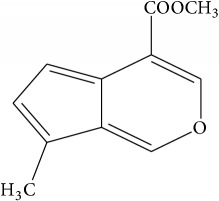

5	14.502	1.688	Cinnamic acid	C_9_H_8_O_2_	148	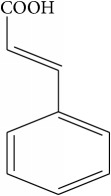

6	15.556	1.987	Phytol	C_20_H_40_O	296	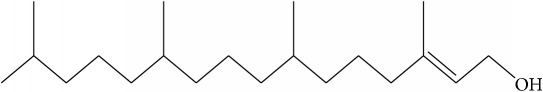

7	16.810	6.643	Palmitic acid	C_16_H_32_O_2_	256	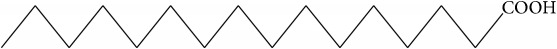

8	16.984	7.202	Geranyl geraniol	C_20_H_34_O	290	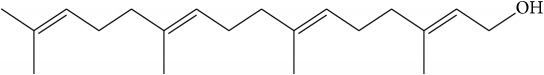

9	18.491	4.315	Oleic acid	C_18_H_34_O_2_	282	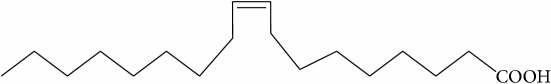

10	18.700	2.514	Stearic acid	C_18_H_36_O_2_	284	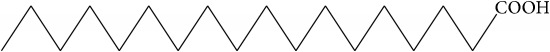

11	22.227	1.461	Eugenol	C_10_H_12_O_2_	164	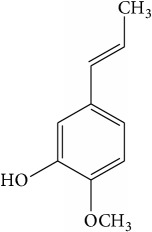

## References

[1] Chaudhari K., Rizvi S., Syed B. A. (2016). Rheumatoid arthritis: Current and future trends. *Nature Reviews Drug Discovery*.

[2] Gabriel S. E., Michaud K. (2009). Epidemiological studies in incidence, prevalence, mortality, and comorbidity of the rheumatic diseases. *Arthritis Research and Therapy*.

[3] Kumar P., Banik S. (2013). Pharmacotherapy options in rheumatoid arthritis. *Clinical Medicine Insights: Arthritis and Musculoskeletal Disorders*.

[4] Vivar N., van Vollenhoven R. F. (2014). Advances in the treatment of rheumatoid arthritis. *F1000Prime Reports*.

[5] Zhang Z.-C., Zhang S.-J., Jin B. (2015). Ciclamilast ameliorates adjuvant-induced arthritis in a rat model. *BioMed Research International*.

[6] Cai Y., Yuan Q., Xu K. (2016). Assessment of the therapeutic effect of total glucosides of peony for juvenile idiopathic arthritis: a systematic review and meta-analysis. *Evidence-Based Complementary and Alternative Medicine*.

[7] Scott D. L., Wolfe F., Huizinga T. W. J. (2010). Rheumatoid arthritis. *The Lancet*.

[8] Crofford L. J. (2013). Use of NSAIDs in treating patients with arthritis. *Arthritis Research & Therapy*.

[9] Grover H. S., Gaba N., Gupta A., Marya C. M. (2011). Rheumatoid arthritis: a review and dental care considerations. *Nepal Medical College Journal: NMCJ*.

[10] Venkatesha S. H., Rajaiah R., Berman B. M., Moudgil K. D. (2011). Immunomodulation of autoimmune arthritis by herbal CAM. *Evidence-Based Complementary and Alternative Medicine*.

[11] Bliddal H., Christensen R., Højgaard L. (2014). Spiritual healing in the treatment of rheumatoid arthritis: an exploratory single centre, parallel-group, double-blind, three-arm, randomised, sham-controlled trial. *Evidence-Based Complementary and Alternative Medicine*.

[12] Siddiqui I., Anis M., Jahan A. A. (2006). Rapid multiplication of Nyctanthes arbor-tristis through in-vitro auxillary shoots proliferation. *World Journal of Agricultural Science*.

[13] Rout G. R., Mahato A., Senapati S. K. (2007). In vitro clonal propagation of *Nyctanthes arbor-tristis* Linn.—a medicinal tree. *Horticulture Science (Prague)*.

[14] Agrawal J., Pal A. (2013). *Nyctanthes arbor*-*tristis* Linn—a critical ethnopharmacological review. *Journal of Ethnopharmacology*.

[15] Gulshan B., Suri K. A., Parul G. (2015). A comprehensive review on *Nyctanthes arbortristis*. *International Journal of Drug Delivery and Research*.

[16] Janbaz K. H., Shabbir A., Mehmood M. H., Gilani A. H. (2014). Pharmacological basis for the medicinal use of *rhus coriaria* in hyperactive gut disorders. *Bangladesh Journal of Pharmacology*.

[17] Kim W., Park S., Choi C. (2016). Evaluation of anti-inflammatory potential of the new ganghwaljetongyeum on adjuvant-induced inflammatory arthritis in rats. *Evidence-Based Complementary and Alternative Medicine*.

[18] Das S., Basu S. P., Sasmal D. (2006). Anti-inflammatory activity of the different parts of *Nyctanthes arbortristis* Linn. *Ethiopian Pharmaceutical Journal*.

[19] Kakoti B. B., Pradhan P., Borah S., Mahato K., Kumar M. (2013). Analgesic and anti-inflammatory activities of the methanolic stem bark extract of *Nyctanthes arbor-tristis* Linn.. *BioMed Research International*.

[20] Shabbir A., Shahzad M., Ali A., Zia-ur-Rehman M. (2016). Discovery of new benzothiazine derivative as modulator of pro- and anti-inflammatory cytokines in rheumatoid arthritis. *Inflammation*.

[21] Shabbir A., Shahzad M., Ali A., Zia-Ur-Rehman M. (2014). Anti-arthritic activity of *N*′-[(2,4-dihydroxyphenyl)methylidene]-2- (3,4-dimethyl-5,5-dioxidopyrazolo[4,3-c][1,2]benzothiazin-1(4*H*)-yl) acetohydrazide. *European Journal of Pharmacology*.

[22] Sathiya M., Parimala P., Muthuchelian K. (2008). Preliminary phytochemical screening and antibacterial studies on the ethanolic leaf extract of *Nyctanthes arbortristis* Linn. *Ethnobotanical Leaflets*.

[23] Vyas A., Sarin R. (2013). Analysis of the phytochemical content and antimicrobial activity of *Nyctanthes arbor- tristis*. *International Journal of Pharma and Bio Sciences*.

[24] Sinha V. S., Ram S. (2015). Qualitative phytochemical analysis of some plants use to cure Malaria in Kolhan region, of Jharkhand. *India. Journal of Medicinal Plants Studies*.

[25] Goyal S., Sheth N., Srivastava D. N. (2013). Comparative evaluation of *Nyctanthes arbortristis* and *Alstonia scholaris* leaves extracts in Freunds complete adjuvant induced arthritis in rats. *International Journal of Pharmaceutical & Biological Archives*.

[26] Reddy V. J. S., Rao G. D., Lakshmi G. R. (2014). A review on anti-arthritic activity of some medicinal plants. *Journal of Global Trends in Pharmaceutical Sciences*.

[27] Patil K. R., Patil C. R., Jadhav R. B., Mahajan V. K., Patil P. R., Gaikwad P. S. (2011). Anti-arthritic activity of bartogenic acid isolated from fruits of *Barringtonia racemosa* Roxb. (Lecythidaceae). *Evidence-Based Complementary and Alternative Medicine*.

[28] McInnes I. B., Schett G. (2011). The pathogenesis of rheumatoid arthritis. *The New England Journal of Medicine*.

[29] Ekambaram S., Perumal S. S., Subramanian V. (2010). Evaluation of antiarthritic activity of *Strychnos potatorum* Linn seeds in Freund's adjuvant induced arthritic rat model. *BMC Complementary and Alternative Medicine*.

[30] Mellado M., Martínez-Muñoz L., Cascio G., Lucas P., Pablos J. L., Rodríguez-Frade J. M. (2015). T cell migration in rheumatoid arthritis. *Frontiers in Immunology*.

[31] Agarwal V., Sachdev A., Lehl S., Basu S. (2004). Unusual haematological alterations in rheumatoid arthritis. *Journal of Postgraduate Medicine*.

[32] Silva R. O., Sousa F. B., Damasceno S. R. (2014). Phytol, a diterpene alcohol, inhibits the inflammatory response by reducing cytokine production and oxidative stress. *Fundamental Clinical Pharmacology*.

[33] Huang X., Liu Y., Lu Y., Ma C. (2015). Anti-inflammatory effects of eugenol on lipopolysaccharide-induced inflammatory reaction in acute lung injury via regulating inflammation and redox status. *International Immunopharmacology*.

[34] Held S., Schieberle P., Somoza V. (2007). Characterization of *α*-terpineol as an anti-inflammatory component of orange juice by in vitro studies using oral buccal cells. *Journal of Agricultural and Food Chemistry*.

[35] de las Heras B., Hortelano S. (2009). Molecular basis of the anti-inflammatory effects of terpenoids. *Inflammation & Allergy-Drug Targets*.

[36] de Santana Souza M. T., Almeida J. R. G. D. S., de Souza Araujo A. A. (2014). Structure-activity relationship of terpenes with anti-inflammatory profile—a systematic review. *Basic and Clinical Pharmacology and Toxicology*.

[37] Tuntiwachwuttikul P., Rayanil K., Taylor W. C. (2003). Chemical constituents from the flowers of *Nyctanthes arbor-tristis*. *ScienceAsia*.

[38] Srivastava V., Rathore A., Ali S. M., Tandon J. S. (1990). New benzoic esters of loganin and 6*β*-hydroxyloganin from *Nyctanthes arbor-tristis*. *Journal of Natural Products*.

[39] Stuppner H., Müller E. P., Mathuram V., Kundu A. B. (1993). Iridoid glycosides from *Nyctanthes arbor-tristis*. *Phytochemistry*.

[40] Nagatoshi M., Terasaka K., Nagatsu A., Mizukami H. (2011). Iridoid-specific glucosyltransferase from *Gardenia jasminoides*. *Journal of Biological Chemistry*.

[41] Viljoen A., Mncwangi N., Vermaak I. (2012). Anti-inflammatory iridoids of botanical origin. *Current Medicinal Chemistry*.

